# miRNA-dependent regulation of STIM1 expression in breast cancer

**DOI:** 10.1038/s41598-019-49629-5

**Published:** 2019-09-10

**Authors:** Rashmi P. Kulkarni, Asha Elmi, Ethel Alcantara-Adap, Satanay Hubrack, Nancy Nader, Fang Yu, Maya Dib, Vimal Ramachandran, Hani Najafi Shoushtari, Khaled Machaca

**Affiliations:** 1 Department of Physiology and Biophysics, Weill Cornell Medicine Qatar, Qatar Foundation, Doha, Qatar; 2 Calcium Signaling Group, Weill Cornell Medicine Qatar, Doha, Qatar; 3 College of Health and Life Sciences, Hamad bin Khalifa University, Qatar Foundation, Doha, Qatar; 4 Department of Cell biology, Weill Cornell Medicine Qatar, Doha, Qatar; 5 Present Address: Managing Partner, Integrated Group, P.O. Box 47039, Doha, Qatar; 6 Present Address: SIDRA Medicine, Doha, Qatar

**Keywords:** Breast cancer, miRNAs

## Abstract

Store-operated Ca^2+^ entry (SOCE) has been shown to be important for breast cancer metastasis in xenograft mouse models. The ER Ca^2+^ sensor STIM1 and Orai plasma membrane Ca^2+^ channels molecularly mediate SOCE. Here we investigate the role of the microRNA machinery in regulating STIM1 expression. We show that STIM1 expression is regulated post-transcriptionally by the miRNA machinery and identify miR-223 and miR-150 as regulators of STIM1 expression in the luminal non-aggressive MCF7 breast cancer cell line. In contrast, STIM1 expression in the more aggressive basal triple-negative MDA-MB-231 cell line is not significantly modulated by a single miRNA species but is rather upregulated due to inhibition of the miRNA machinery through downregulation of Ago2. Consistently, overexpression of Ago2 results in decreased STIM1 protein levels in MDA-MB-231 cells. Clinically, STIM1 and Ago2 expression levels do not correlate with breast cancer progression, however in the basal subtype high STIM1 expression is associated with poorer survival. Our findings show that STIM1 expression is differentially regulated by the miRNA machinery in different cell types and argue for a role for this regulation in breast cancer.

## Introduction

Breast cancer is the most common cancer in women representing the leading cause of cancer deaths in this gender and accounts for ~25% of all cancer cases^1^. Notwithstanding significant advances in breast cancer treatment ~30% of patients relapse resulting in a death toll of >450,000 annually^1^. Over 90% of the mortality associated with breast cancer is due to metastatic disease and not the primary tumor^2^. Metastasis in a multistep complex process that involves cells in the primary tumor loosening their cell-cell contact with adjacent cells, migrating to the lymphatic or blood circulation, infiltrating the circulatory system, penetrating the target tissue through the capillary endothelium, and colonizing and surviving is the secondary site^3^. Cell migration requires the formation of membrane protrusions at the front edge of the cell and detachment at the back end, both of which being Ca^2+^-dependent processes. Furthermore, store-operated Ca^2+^ entry (SOCE) has been implicated in modulating the actin cytoskeleton and cell mobility^4,5^.

SOCE is mediated by the STIM and Orai family of proteins. STIM1 is a Ca^2+^ sensor that senses Ca^2+^ load in the ER and forms clusters in response to store depletion that localize to ER-plasma membrane (PM) junctions where they recruit and gate open the highly Ca^2+^ selective channel Orai1^6^. The deregulation of SOCE has been associated with a variety of diseases^7^, including breast cancer. STIM1 and Orai1 have been shown to be required for breast cancer metastasis in xenograft models^5^. The role of SOCE has been further extended to migration and metastasis of other cancers, including cervical^8^, melanoma^9^, and colorectal cancer^10^. Interestingly, STIM1 (GOK) was initially identified as a tumor-suppressor^11^. These findings support a central role for SOCE in cancer metastasis.

MicroRNAs (miRNAs) are endogenous single-stranded RNAs (21–24 nucleotides) that function in post-transcriptional regulation of gene expression^12^. miRNAs typically recognize target genes through their seed sequence (2–7 nt) binding at the 3′ untranslated regions (3′UTR). This leads to target mRNA degradation and/or inhibition of translation^12–15^. Any given 3′UTR may have multiple binding sites for a number of different miRNAs. Conversely, any single miRNA can bind to 3′UTRs of several target genes. This mechanism allows precise regulation of genes by several miRNAs as well as co-regulation of genes by a single miRNA^16^. The majority of cytoplasmic miRNAs are bound to Argonaut protein2 (Ago2) and form an effector protein complex, the RNA-induced silencing complex (RISC), that targets mRNAs for degradation or regulates translation^17^.

Herein, we show that STIM1 expression is differentially regulated by the microRNA machinery in non-aggressive luminal MCF7 cells as compared to the aggressive basal MDA-MB-231 cells. STIM1 is maintained at low levels in MCF7 by miR-223 and miR-150 among other miRNAs. In contrast, the high levels of STIM1 expression in MDA-MB-231 cells can be explained by a global downregulation of the miRNA machinery through a reduction in Ago2 expression.

## Results

### STIM1 expression is regulated at the post-transcriptional level

We were first interested in assessing whether STIM1 expression is regulated as the post-transcriptional level in breast cancer cell lines. We chose two canonical cell lines: the basal, aggressive, triple negative MDA-MB-231 line and the luminal, hormone positive MCF-7 line, because they are extensively used as prototypical examples of aggressive and non-aggressive breast cancers and importantly because they have been shown to express STIM1 at significantly different levels^5,18^.

**Figure 1:**
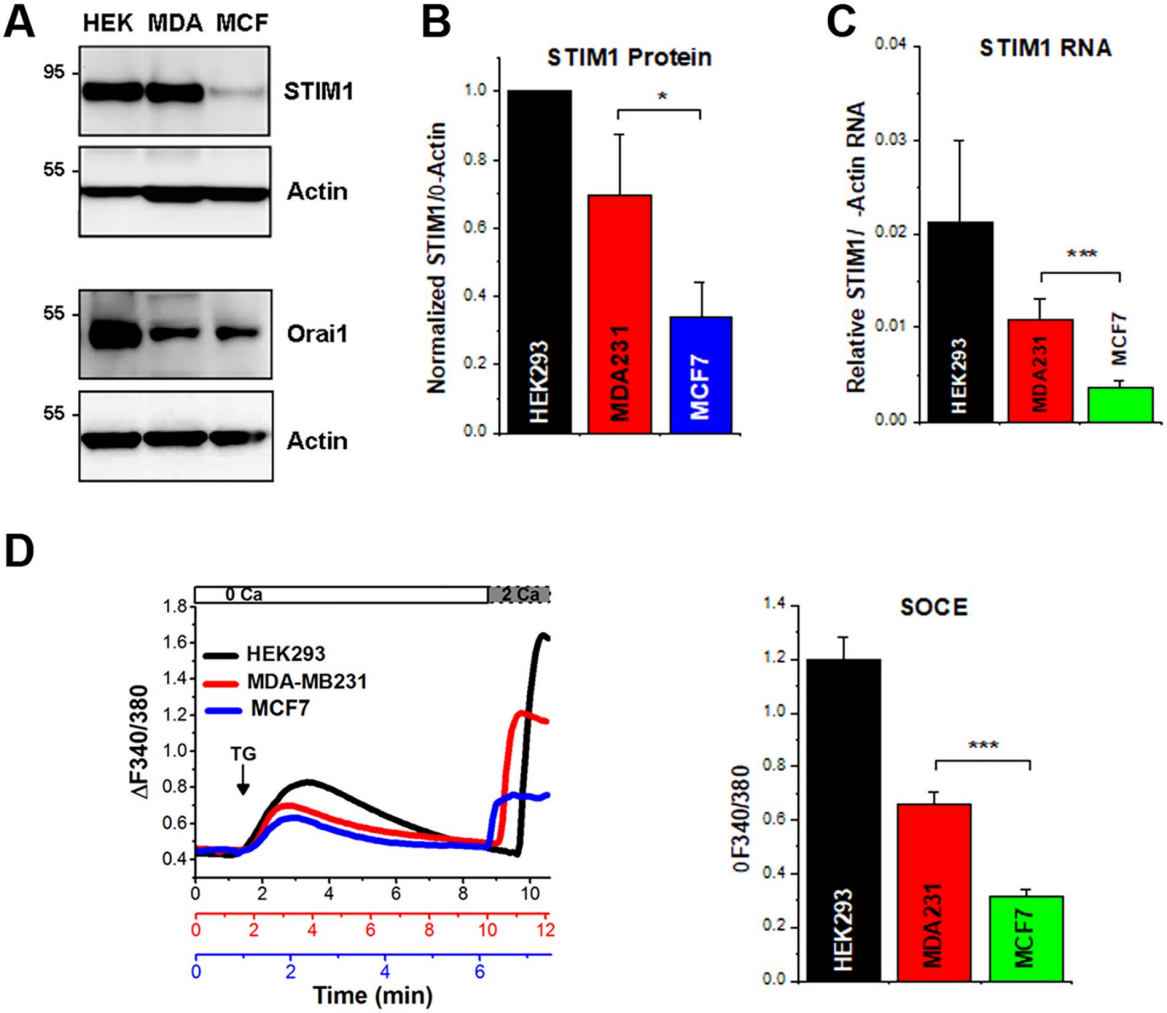
STIM1 expression differentially regulates SOCE levels in MCF7 and MDA-MB-231 cells. (**A**) Representative Western blots to assess STIM1 and Orai1 protein levels in HEK-293, MDA-MB231 and MCF7 cells with β-actin as a loading control. (**B**) Quantification of STIM1 protein levels as the ratio of STIM1/actin (mean ± S.D.; ANOVA; p = 0.04, n = 3). (**C**) STIM1 mRNA levels assessed by RT-PCR in HEK-293, MCF7, and MDA-MB231 cells (mean ± S.D.; ANOVA; p < 0.0001; n = 6). (**D**) SOCE levels measured in the three cell lines loaded with Fura-2 and with store depletion induced with thapsigargin (TG, 1 µM) in Ca^2+^-free medium (0Ca) followed by Ca^2+^ addition (2Ca; 2 mM). Example traces (left) and quantification of SOCE levels (right) (mean ± S.D.; ANOVA, p < 0.0001; n = 3–4).

We confirmed the significantly higher STIM1 expression in MDA-MB-231 as compared to MCF-7 cells both at the protein (Fig. 1A,B) and mRNA levels (Fig. 1C). In contrast to STIM1, both cell lines express similar levels of Orai1 (Fig. 1A). We used HEK-293 cells throughout these studies as a non-breast cancer cell line as it has been used extensively to study SOCE. HEK-293 cells express high levels of both STIM1 and Orai1 (Fig. 1A,B). STIM1 protein expression correlates with steady-state STIM1 transcript levels in the three cell lines (Fig. 1B,C). To test the effects of differential STIM1 expression on SOCE, we measured SOCE using the standard thapsigargin-mediated store depletion followed by Ca^2+^ re-addition protocol, to temporally separate Ca^2+^ release from SOCE (Fig. 1D). SOCE levels were significantly lower in MCF-7 cells compared to MDA-MB-231, with HEK-293 cells exhibiting the highest SOCE (Fig. 1D). These data show that SOCE levels correlate with STIM1 expression.

### The STIM1 3′UTR regulates translation in different cell types

**Figure 2:**
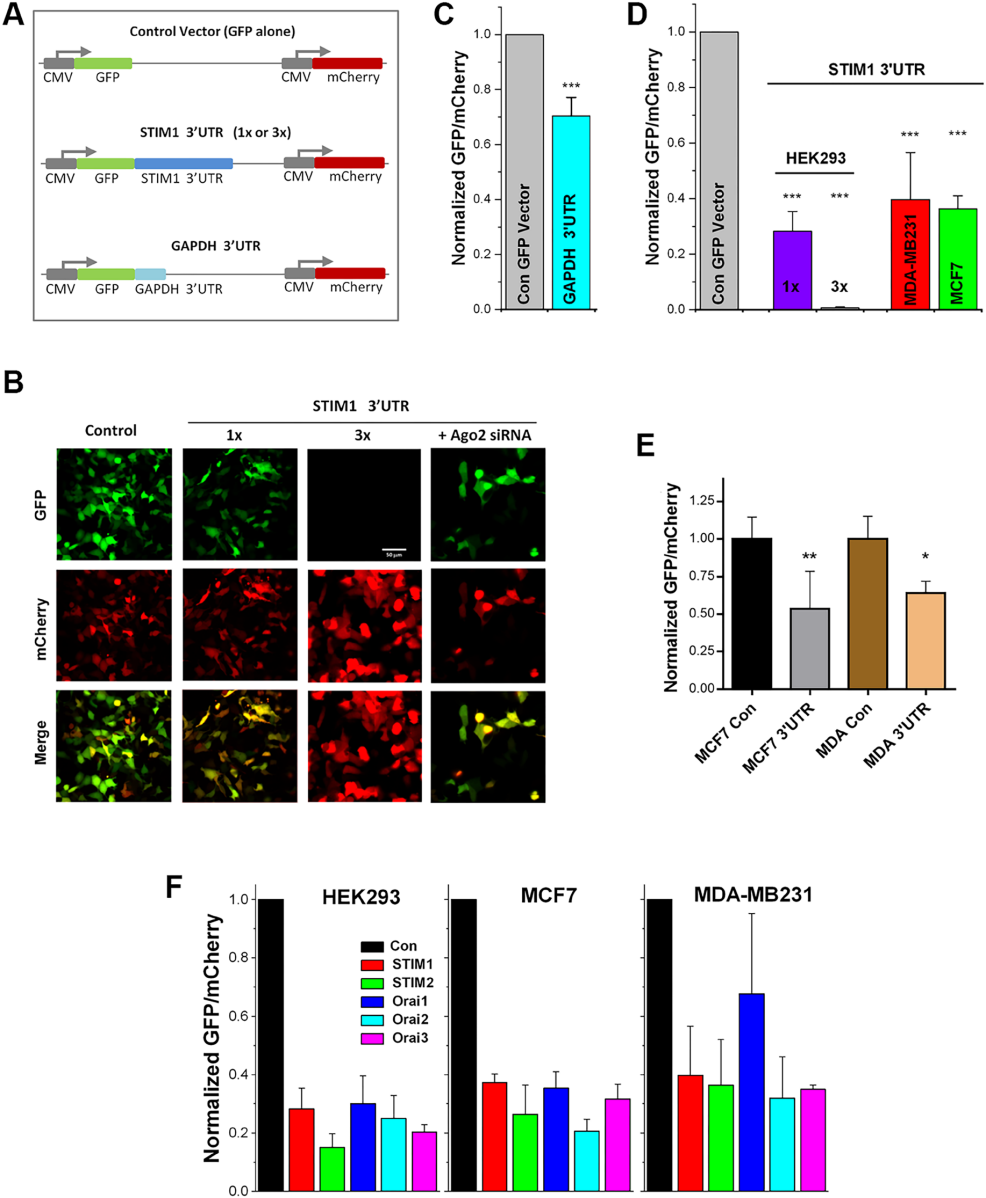
The STIM1 3′UTR regulates STIM1 expression. (**A**) Cartoon of the different GFP-mCherry reporter constructs used to study post-transcriptional regulation mediated by the 3′UTRs. (**B**) Representative images of HEK-293 cells expressing the control vector (Control), or GFP followed by one (1x) or three copied (3x) of the STIM1 3′UTR. Also shown are images from cells expressing STIM1 3′UTR 1x where Ago2 has been knocked-down (Ago2 siRNA). (**C**) Normalized GFP/mCherry expression ratios in HEK293 cells expressing the control vector or the GAPDH 3′UTR vector. (**D**) Normalized GFP/mCherry expression ratios were assessed by single cell imaging and normalized to the average ratio for the control vector in each cell line studied. HEK-293 cells were transfected with the control vector, STIM1 3′UTR (1x or 3x). MCF7 and MDA-MB-231 cells were transfected with the control vector or the STIM1 3′UTR (1x). The data were normalized to the control vector for each cell line. Data are mean ± S.D.; ANOVA, p < 0.0002, n = 3–4). (**E**) Normalized GFP/mCherry intensity in MCF7 and MDA-MB321 cells overexpressing the control vector or the STIM1 3′UTR construct as analyzed by flow cytometry (mean ± S.D.; paired t-test; **p = 0.0046; *p = 0.0202; n = 3). (**F**) Ratio of the GFP/mCherry reporter expression from HEK-293, MDA-MB231 and MCF7 cells transfected with 3′UTR constructs of STIM1, STIM2, Orai1, Orai2 and Orai3.

To test whether STIM1 expression is regulated post-transcriptionally through its 3′UTR, we engineered a reporter construct that allows single cell ratiometric analysis of 3′UTR-dependent regulation of protein expression. GFP and mCherry were expressed downstream of the CMV promoter in the same vector as a control (Fig. 2A), to allow for similar expression levels of both fluorescent proteins. Assessing the ratio of GFP/mCherry allows for single cell quantification of steady state protein levels. The GAPDH-3′UTR construct was used as a control (Fig. 2A). Compared to the control vector, insertion of the GAPDH 3′UTR downstream of the GFP coding region resulted in a significant downregulation of GFP expression (Fig. 2C). The STIM1-3′UTR significantly and more effectively downregulated GFP expression in a dose dependent, and including three copies of the STIM1 3′UTR in tandem after GFP (Stim1 3′UTR 3X) almost completely eliminates GFP expression (Fig. 2B,D). Similar to HEK293 cells, the STIM1 3′UTR regulates protein expression in both the MDA-MB-231 and the MCF7 cell lines (Fig. 2D). We further confirmed the role of the STIM1 3′UTR in regulating protein expression in MCF7 and MDA-MB-231 cells using flow cytometry to better assess their effects on a larger cohort of transfected cells (Fig. 2E). Consistent with the single cell imaging data, the STIM1 3′UTR downregulates GFP expression in both cell lines (Fig. 2E). Together these data show that STIM1 3′UTR contributes to the regulation of STIM1 expression.

We then tested using the GFP-mCherry reporter whether the 3′UTR of other members of the STIM and Orai families downregulate protein expression in a similar fashion to the STIM1 3′UTR. Figure 2F shows that the 3′UTR of all five members of STIM and Orai families result in decrease GFP expression in HEK293, MCF7 and MDA-MB-231 cells. This shows that the 3′UTR of the STIM and Orai gene family affords post-transcriptional downregulation of protein expression. However, the level of downregulation is cell type specific, with the 3′UTR of Orai1 showing the most variability where it results in lower levels of downregulation in MDA-MB-231 cells as compared to HEK293 or MCF7 cells (Fig. 2F).

### STIM1 3′UTR-mediated regulation is Ago2-dependent

**Figure 3:**
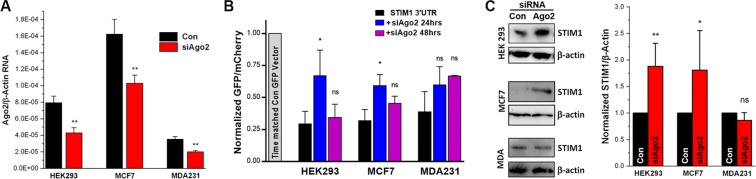
STIM1 3′UTR-mediated downregulation of expression requires Ago2. (**A**) RT-PCR quantification of Ago2 message levels after transfection with the control siRNA or siRNA targeting Ago2 in HEK-293, MCF7 and MDA-MB231 cells (mean ± S.D.; unpaired t-test; p ≤ 0.007; n = 3). (**B**) Expression of the GFP-mCherry reporter in cells transfected with the control vector (Control GFP vector) or the STIM1 3′UTR construct without siRNA treatment or after Ago2 siRNA transfection at 24 and 48 hours. Data are normalized to the control vector in each cell line (mean ± S.D.; ANOVA; *p ≤ 0.0406; ns p = 0.0697; n = 3). (**C**) Representative Western blots showing STIM1 expressions with (Ago2) or without (Con) Ago2 knockdown at 24 hours in HEK-293, MCF7 and MDA-MB231 cells (left) and quantification data of the ratio of STIM1/actin in the three cell lines (right) (mean ± S.D.; ratio paired t-test; **p = 0.0052; * 0.0371; n = 3–5).

The above results suggest that the STIM1 3′UTR regulates expression through the miRNA machinery. To test whether this is the case we knocked-down Ago2 expression using siRNA (Fig. 3A). We confirmed the effectiveness of Ago2 knock-down by RT-PCR in HEK293, MCF7 and MDA-MB-231 cells (Fig. 3A). Notably, we found that the basic levels of Ago2 mRNA were significantly different between MCF7 and MDA-MB-231 cells (Fig. 3A, black bars). We then used the GFP-mCherry reporter to assess the effects of Ago2 downregulation on the inhibitory effects of the STIM1 3′UTR on protein expression. siRNA against Ago2 reversed the inhibitory effects of the STIM1 3′UTR on GFP expression in HEK293 and MCF7 cells (Fig. 3B), and this effect was lost at 48 hrs post siRNA transfection (Fig. 3B).

The response to Ago2 knockdown was different in MDA-MB-231 cells, where although there was a trend for reversing the inhibitory effect of the STIM1 3′UTR on GFP expression, the data did not reach statistical significance at either the 24 or 48 hrs time points (Fig. 3B). This argues that STIM1 expression in HEK293 and MCF7 is Ago2-dependent but to a lesser extent in MDA-MB-231 cells at endogenous Ago2 expression levels. This is consistence with significantly lower levels of Ago2 mRNA in MDA-MB-231 compared to MCF7 and HEK293 (Fig. 3A, black bars).

Since the STIM1 3′UTR regulates the expression of the reporter in HEK293 and MCF7 cells but to a lesser extent in MDA-MB-231 cells, we wanted to determine whether the Ago2/miRNA pathway regulates endogenous STIM1 levels in a similar fashion. We knocked-down Ago2 using the siRNA approach and measured STIM1 protein levels (Fig. 3C). Twenty four after Ago2 siRNA treatment, endogenous STIM1 expression is significantly up-regulated in HEK 293 and MCF7 (Fig. 3C). This validates the use of the reporter construct as a marker for endogenous STIM1 expression, with the caveat that the reporter is likely to be expressed at higher levels than endogenous STIM1 following transient transfection, and as such may be more sensitive to regulation by the miRNA machinery. In contrast to HEK293 and MCF7 cells, knockdown of Ago2 in MDA-MB-231 cells (Fig. 3A), did not alter STIM1 protein levels (Fig. 3C). This shows that endogenous STIM1 expression is less dependent on the miRNA machinery in MDA-MB-231 cells.

To further functionally test the effects of endogenous STIM1 expression on SOCE in the breast cancer cell lines, we measured SOCE levels as in Figure 1 in MCF7 and MDA-MB-231 cells following overexpression of STIM1 protein (Supp. Fig. S1A,B). STIM1 overexpression in MDA-MB-231 cells does not significantly alter SOCE levels (Supp. Fig. S1A), presumably because STIM1 is not limiting in these cells, as endogenous STIM1 is highly expressed (Fig. 1A–C). In contrast, in MCF7 where STIM1 expression levels are low (Fig. 1A–C), STIM1 overexpression leads to higher Ca^2+^ influx through SOCE (Supp. Fig. S1B). We further reasoned that if the STIM1-3′UTR binds miRNAs to regulate STIM1 levels in MCF7 cells, then overexpression expression of the STIM1 3′UTR alone should act as a sponge and sequester endogenous miRNAs, which would relieve post-transcriptional inhibition of endogenous STIM1 expression. Consistent with this idea, when we overexpressed the STIM1 3′UTR in the context of the GFP-mCherry reporters, either with 1 (1x) or 3 copies of the STIM1 3′UTR in tandem (3x) downstream of the GFP coding region, we observe a concomitant significant increase in SOCE levels with the 3x but not the 1x construct (Supp. Fig. S1C). These results argue that sequestering endogenous miRNAs leads to upregulation of SOCE.

### miR-223 regulates STIM1 expression

**Figure 4:**
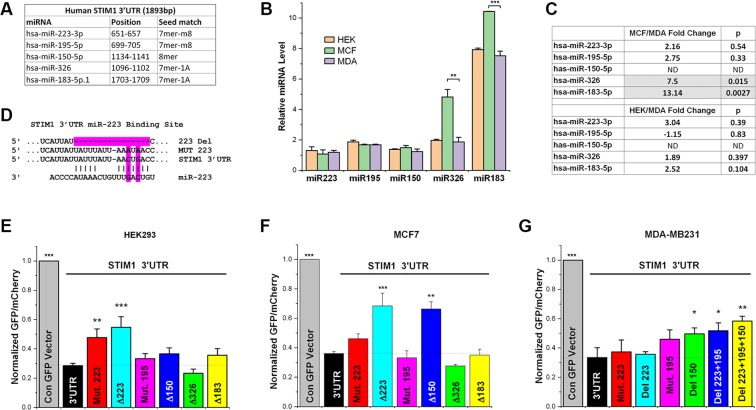
miRNAs differentially regulate STIM1 3′UTR-dependent expression. (**A**) Location of the miRNA binding sites within the STIM1 3′UTR as identified by Targetscan v6.2. The length of the seed match between the miRNA and the 3′UTR is also indicated. (**B**) Expression levels of the different miRNAs determined by miRNA microarray analysis using three biological replicates of total RNA samples (mean ± S.D.; ANOVA; **p = 0.0077; ***p < 0.0001; n = 3). (**C**) Relative expression levels of the different miRNA quantified using RT-PCR with the p-values indicated for each ratio (n = 3). ND indicates not-determined given undetectable levels of the miRNA in one or both of the cell lines. (**D**) Example mutant and deletion clones within the STIM1 3′UTR shown for miR-223 within the STIM1 3′UTR experimental plasmid. (**E–G**) Effect of the different mutant or deletions of the miRNA binding sites within the STIM1 3′UTR on the expression levels of GFP measured using the GFP-mCherry reporter. All statistical analyses were performed relative to the STIM1 3′UTR construct (3′UTR) in the three cell lines as indicated (mean ± S.D.; ANOVA; *p ≤ 0.0418; **p ≤ 0.0038; ***p < 0.0001; n = 3–8).

To directly identify potential miRNAs that regulate STIM1 post-transcriptionally, we used TargetScan (v 6.2)^19,20^, which identified miR-223, miR-195 and miR-150 with binding sites in the STIM1 3′UTR as broadly conserved among vertebrates (Fig. 4A). This *in silico* identification of miRNA binding sites is partially consistent with the experimental identification of miR-195, in coordination with the RNA-binding protein HuR, as regulators of STIM1 expression in intestinal epithelial cells (IECs)^21^. We reasoned that the differential expression of STIM1 in MCF7 as compared to MDA-MB-231 cells could be due to differential expression of miRNAs that target the STIM1-3′UTR. We used miRNA microarrays to determine the levels of expression of miRNAs predicted to target the STIM1 3′UTR in HEK293, MDA-MB231 and MCF7 cells. The levels of the three conserved miRNAs (miR-223, miR-195, and miR-150) were similar in the three cell lines (Fig. 4B), suggesting that this cannot account for the differential STIM1 expression. We then looked for miRNAs that target the STIM1-3′UTR, albeit with poor conservation among vertebrates, but that are differentially expressed in MDA-MB-231 versus MCF7 cells. This analysis identified miR-326 and miR-183 with higher expression in MCF7 compared to MDA-MB-231 cells (Fig. 4B), which would be expected to downregulate STIM1 expression in MCF7 cells. We further validated the microarray data using a more quantitative real-time PCR approach, which confirmed the trends observed with the microarray approach (Fig. 4C). PCR quantification shows that miR-223, miR-195 and miR-150 are not differentially expressed in the three cell lines (Fig. 4C). In contrast, miR-326 and miR-183 are significantly upregulated (7.5 and 13 folds respectively) in MCF7 cells as compared to MDA-MB-231 cells (Fig. 4C).

Despite the variability observed in miRNA levels, we were interested in directly testing the role of these individual miRNAs on STIM1 expression. We used the GFP-mCherry reporter with the STIM1 3′UTR as the control and introduced either point mutations to alter two core bases within the seed of the individual miRNAs of interest, or deleted the entire region in the 3′UTR that shows complementarity to the miRNA in question as shown for miR-223 as an example (Fig. 4D). The different mutant and deletion constructs were tested in HEK293, MCF7, and MDA-MB-231 cells to assess whether there is a correlation between the miRNA expression profiles and STIM1 levels (Fig. 4E–G).

In HEK293 cells only alterations to the miR-223 binding site resulted in a rescue of the GFP expression inhibition mediated by the STIM1 3′UTR, while deletions or mutations to the miR-195, miR-150, miR-326 and miR-183 had no effect (Fig. 4E). Deletion of the miR-223 binding site, but not mutating it, also rescued the STIM1 3′UTR-mediated inhibition of expression in MCF7, as well as deletion of the miR-150 binding site (Fig. 4F). This argues for an important role for both miR-223 and miR-150 in regulating STIM1 expression in MCF7 cells. Surprisingly though deleting the binding sites for miR-326 or miR-183, both of which are expressed at higher levels in MCF7 cells compared to MDA-MB-231 cells, had no effect (Fig. 4F). This argues that differential miRNA expression in MCF7 vs MDA-MB-231 cells is unlikely to regulate the expression of STIM1.

The response to miRNA binding sites deletions or mutations was disparate in MDA-MB-231 cells (Fig. 4G). We did not detect any rescue of STIM1 3′UTR-mediated inhibition after deleting or mutating the miR-223 or miR-195 binding sites, and only a relatively small although statistically significant rescue in response to deleting the miR-150 binding site (Fig. 4G). The level of rescue and significance increased as we combined multiple deletions of the conserved miRNAs binding sites with the most significant effect observed in response to deleting the binding sites for miR-223, miR-195 and miR-150 (Fig. 4G). These results suggest that the miRNA-directed inhibition of gene expression in MDA-MB-231 cells is less effective than in MCF7 cells.

### Distinct cell-type specific miRNA-dependent mechanisms regulate STIM1 expression

**Figure 5:**
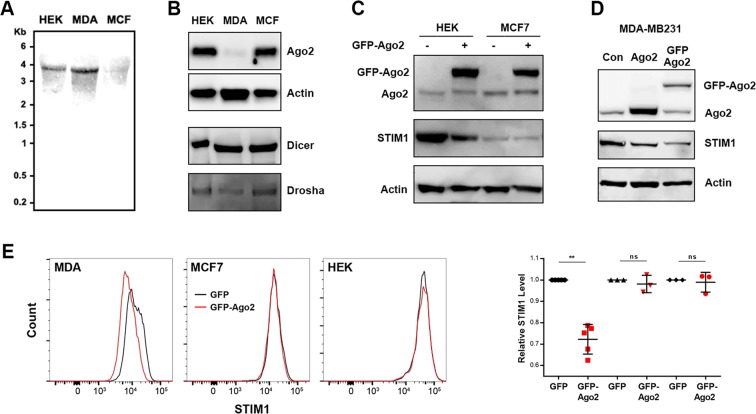
Ago2 expression is downregulated in MDA-MB-231 cells. (**A**) Northern blotting with DIG-labeled STIM1 coding region probe to asses STIM1 transcripts in the three cell lines. (**B**) Western blot showing endogenous protein expression of Ago2, Dicer, and Drosha in HEK-293, MDA-MB231, and MCF7 cells. Actin is shown as a loading control. Representative of three or more similar gels. (**C**) STIM1 levels in HEK293 and MCF7 overexpressing GFP-Ago2. Actin is shown as a loading control. Representative of three similar experiments. (**D**) Western blots showing endogenous Ago2, untagged Ago2, tagged Ago2, actin and STIM1 levels in MDA-MB231 cells overexpressing untagged and GFP-tagged Ago2. Representative of 5 similar experiments. (**E**) Overexpression of Ago2 results in decreased STIM1 protein levels in MDA-MB-231 cells. Representative histograms (left) and summary data right) of flow cytometry analysis of endogenous STIM1 expression levels from GFP or GFP-Ago2 transfected MDA-231, MCF7 and HEK293 cells (Live GFP^+^ cells) (Mean ± S.D.; ratio paired t-test; **p = 0.0017; ns: not significant; n = 3–5).

The above results argue that differential expression of miRNAs targeting the STIM1 3′UTR is unlikely to mediate the higher STIM1 expression in MCF7 compared to MDA-MB-231 cells. We were thus interested in other mechanisms that could differentially regulate STIM1 expression by targeting its 3′UTR. One potential mechanism is 3′UTR shortening, which has been documented during T cell activation^22^, neuronal activation^23^ and in cancer cells^24^. Such 3′UTR shortening in MDA-MB-231 cells would relieve the miRNA dependent inhibition, which would be expected to increase STIM1 expression. We thus tested for 3′UTR shortening in MDA-MB-231 using Northern blotting. We detected STIM1 transcripts using a probe within the STIM1 coding region and found that all three cell lines express the full-length 4 kb transcript (Fig. 5A). No smaller transcripts were detected in the MDA-MB231 that would corroborate the 3′UTR clipping hypothesis. Consistent with the RT-PCR data the level of the STIM1 message in MCF7 was much reduced (Fig. 5A).

Another potential mechanism that could regulate miRNA-dependent post-transcriptional regulation is through the Pumilio family, which has been shown to bind to mRNAs and modulate their structure to expose otherwise inaccessible miRNA binding sites^25^. Interestingly, the STIM1 3′UTR has a potential Pumilio binding site 604-UGUACAUA-611, that matches the highly conserved 8-nt core motif UGUA(A/U/C)AUA for known Pumilio binding sites^26^. Therefore Pumilio proteins could be inhibiting miRNA interactions with the STIM1 3′UTR in MDA-MB-231 cells leading to increased STIM1 expression. To test this possibility we knocked-down both Pumilio isoforms using siRNA and tested the effect on STIM1 expression in MDA-MB-231 cells (Supp. Fig. S2). siRNA treatment effectively knocked-down PUM1 and PUM2 expression but did not consistently affect STIM1 expression (Supp. Fig. S2), ruling out Pumilio-mediated regulation of miRNA binding as a mechanism controlling STIM1 expression in MDA-MB-231 cells.

We then turned to the miRNA machinery and tested for the levels of expression of Ago2, Dicer and Drosha, three proteins required for miRNA maturation and targeting^27^. Dicer and Drosha are enzymes that clip and process the primary transcribed miRNA into the mature miRNA that is loaded on the RISC complex containing Ago2 for targeting specific mRNA species. We did not detect any changes in Dicer or Drosha expression in HEK-293, MCF7 and MDA-MB-231 cells (Fig. 5B). This is consistent with the microarray and RT-PCR miRNA data (Fig. 4B,C) showing no global alterations in miRNA expression among the three cell lines.

In contrast, Ago2 levels were dramatically reduced in MDA-MB-231 cells compared to MCF7 or HEK-293 cells (Fig. 5B). This argues that the miRNA effector function in the metastatic MDA-MB-231 cells is inferior to that in the non-metastatic MCF7 cells. To test whether this downregulation of Ago2 could affect STIM1 protein levels in MDA-MB-231 cells, we overexpressed either untagged or GFP-tagged Ago2 and assessed STIM1 expression (Fig. 5E-F). By Western blot analyses Ago2 overexpression was coupled in most experiments to a significant downregulation of STIM1 levels (Fig. 5D). In contrast, GFP-Ago2 overexpression in MCF7 cells did not alter endogenous STIM1 expression levels (Fig. 5C). However, such experiments on cell populations could be fraught with errors especially in MDA-MB-231 cells which were difficult to transfect in our hands. We therefore resorted to flow cytometry, which affords an analysis at the single cell level, to assess STIM1 expression levels in MDA-MB-231 cells. We transfected cells with GFP-Ago2 to allow gating on the GFP^+^ channel to isolate transfected cells, and stained cells for STIM1 (Fig. 5E). Expression of Ago2 had no effect on STIM1 levels in HEK-293 or MCF7 cells (Fig. 5E). In contrast, in MDA-MB-231 cells Ago2 expression constantly lowered STIM1 expression levels (Fig. 5E). This shows that the higher STIM1 expression in MDA-MB-231 cells is due to downregulation of Ago2. Lower Ago2 levels have been shown to stabilize the inactive miRNA precursors despite dicer activity^28^. This explains the decreased miRNA-dependent regulation of STIM1 in MDA-MB-231 cells despite the lack of alterations in global microRNA expression.

### STIM1 and Ago2 expression in breast cancer subtypes

The results from MCF7 and MDA-MB-231 cells argues for Ago2 downregulation as a mechanism to lower the effectiveness of the miRNA machinery in aggressive breast cancer cells resulting in increased STIM1 expression to support cell migration. Cancers however are notoriously heterogeneous with multiple mechanisms converging to endow cancer cells with their capability to metastasize. We were therefore interested in assessing whether the findings from the two cell lines, MCF7 and MDA-MB-231, translate to the clinical setting with its inherent heterogeneity.

**Figure 6:**
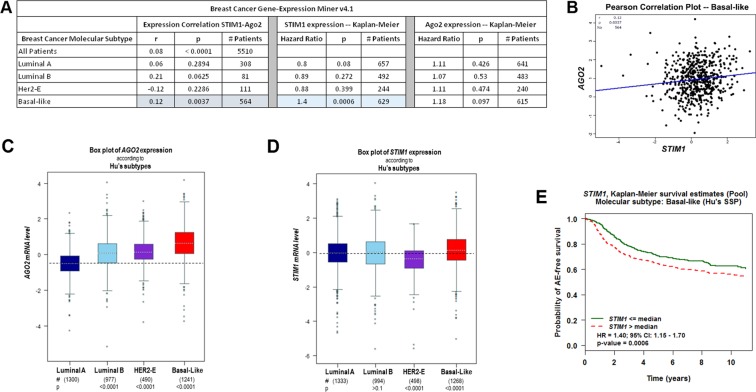
Breast cancer progression, STIM1 and Ago2. All analyses were performed on the Breast Cancer Gene-Expression Miner v4.2 platform (bc-GenExMiner v4.2) using publically available datasets. (**A**) Table outlining the correlation between the expression of STIM1 and Ago2 based on the breast cancer molecular subtype as listed. The correlation coefficient (r), statistical significant (p) and the number of patients used for each analysis are shown. Also shown at the hazard ratios associate with STIM1 and Ago2 expression based on Kaplan-Meier survival curves for each molecular subtype. (**B**) Scatter plot of Ago2 versus STIM1 expression for the basal-like breast cancer molecular subtype. (**C**,**D**) Box plots of mRNA levels for Ago2 (**C**) and STIM1 (**D**) classified based on the breast cancer molecular subtype. Below each subtype classification the number of patients (#) used for the analysis as well as the p-value compared to the luminal A subtype are shown. (**E**) Kaplan-Meier survival curve for patients with basal-like breast cancer subtype with the green curve showing event-free survival for patient with STIM1 levels below or equal to the median and the red-dash curve for patients with STIM1 levels above the median levels.

We resorted to the publicly available datasets through the Breast Cancer Gene Expression Miner (v4.1), and analyzed breast cancers based on their molecular subtype classification going from the mildest luminal A subtype to the most severe aggressive and metastatic basal-like subtype. The two extremes correlate with the MCF7 and MDA-MB-231 cell lines. We first asked whether there exists a correlation between the levels of Ago2 and STIM1 expression to corroborate the findings in the cell lines. The only statistically significant correlation was in the most aggressive basal-like subtype (Fig. 6A,B), but it was a positive weak correlation (r = 0.12), where increased Ago2 levels correlate with higher STIM1 levels (Fig. 6A,B). This argues against Ago2 downregulation as a constant mechanism controlling breast cancer progression clinically. Consistent with this conclusion the levels of Ago2 expression increased with the severity of the breast cancer subtype (Fig. 6C). In contrast, STIM1 levels were more stable across the different molecular subtype and did show significant upregulation in the basal-like subtype (Fig. 6D).

Is this STIM1 upregulation significant in terms of prognosis? To answer this question we plotted survival curves for the different molecular subtypes based on the levels of STIM1 expression. Higher STIM1 expression correlates with an increased hazard ratio (1.4) in the basal-like subtype (Fig. 6E and 6A), but not the other subtypes (Fig. 6A). Therefore, in the aggressive basal-like subtype higher STIM1 correlates with decreased survival, arguing for STIM1 expression as an important modulator of breast cancer progression. This conclusion is consistent with previous reports that showed a correlation between cancer progression and STIM1 expression^29,30^. Furthermore, we did not observed any correlation between the levels of Ago2 expression and survival in any of the breast cancer subtypes (Fig. 1A).

## Discussion

miRNAs have been implicated in regulating SOCE in different tissues. STIM1 expression has been shown to be regulated by miR-195 in intestinal epithelial cells (miR-195)^21^; miR-424 in vascular smooth muscle^31^; miR-185 in colorectal cancer^10^, and miR-223 in cardiac hypertrophy^32^ and glioblastomas^33^.

Here we expand on these findings and show that all members of the STIM and Orai gene families are regulated post-transcriptionally through their 3′UTR. We engineered a fluorescent GFP-mCherry reporter that allows for assessment of post-transcriptional expression regulation using either fluorescence microscopy or flow cytometry (Fig. 2). This reporter is more sensitive to miRNA-dependent regulation than endogenous STIM1, as it shows STIM1 3′UTR-dependent downregulation in MDA-MB-231 (Fig. 2D) whereas Ago2 knockdown in these cells does not affect endogenous STIM1 levels (Fig. 3C). This is likely due to the high levels of expression of the reporter following transient transfection where it would bind more of the endogenous miRNAs and thus exhibit a higher sensitivity than endogenous STIM1. Nonetheless, the reporter offers a convenient methods to screen for miRNAs that regulate protein expression.

The STIM1 3′UTR regulates reporter expression through miR-223 and miR-150 in a cell type specific fashion. miR-223 modulates STIM1 3′UTR-dependent expression in HEK-293 and MCF7 cells but not MDA-MB-231 cells. We further show that the high STIM1 expression in MDA-MB-231 cells is not due to a specific miRNA, rather it reflects a downregulation of Ago2 expression. Consistently overexpression of Ago2 results in lower STIM1 protein levels in MDA-MB-231 cells. The downregulation of Ago2 expression in MDA-MB-231 compared to MCF7 cells was also observed by Fan *et al*., who nonetheless demonstrated that Ago2 is functional in MDA-MB-231 cells as it associate with miRNAs to regulate protein expression^34^.

A recent study explored the role of miR-223 in regulating STIM1 expression in MCF7 and MDA-MB-231^35^. This work argues that miR-223 levels are higher in MCF7 as compared to MDA-MB-231 cells. We were unable to replicate their findings using either non-targeted miRNA microarrays or RT-PCR approaches (Fig. 4). The reasons for the discrepancy are not clear, nonetheless, the fact that Ago2 is dramatically downregulated in MDA-MB-231 cells minimizes the importance of a particular miRNA in regulating STIM1 expression.

Our findings further complement the work of Motiani *et al*. who showed that an Orai isoform switch contributes to the differential SOCE levels in MCF7 vs MDA-MB-231 cells^18^. MCF7 cells not only express low levels of STIM1, but also rely on Orai3, a less efficient Ca^2+^ channel that was shown to be regulated by estrogen receptor α^36^. Despite the differences in Orai isoforms and their efficiency, we found that overexpressing STIM1 caused a significant increase in SOCE in MCF7 cells. This indicates that STIM1 levels regulate SOCE in MCF7 cells.

The regulation of STIM1 expression is not limited to the post-transcriptional level as it has been shown to be regulated transcriptionally as well. STIM1 along with Orai1 transcription are regulated by the HINT1 transcription factor in mouse embryonic fibroblasts and Neuro-A cells^37^. In addition, STIM1 expression in Wilms kidney tumors is regulated at the transcriptional level by the zinc-finger proteins Wilms tumor suppressor 1 and early growth response 1^38^.

Our results show that STIM1 miRNA-dependent post-transcriptional regulation is used effectively to regulate STIM1 expression in two different breast cancer cell lines. Consistently, STIM1 expression correlates with a poorer prognosis in aggressive basal-like breast cancers (Fig. 6). However analysis of clinical data argue against Ago2-dependent STIM1 upregulation as a consistent mechanism supporting breast cancer progression, because even in the most aggressive basal-like subtype there is no negative correlation between Ago2 and STIM1 expression.

In summary, our findings show that Ago2 downregulation could be an effective strategy to upregulate STIM1 expression as observed in MDA-MB-231 cells, suggesting it as one potential mechanism to control STIM1 expression during breast cancer development.

## Materials and Methods

### Cell culture

HEK-293, MDA-MB231 and MCF7 cells were obtained from ATCC. HEK-293 and MDA-MB231 cells were cultured in DMEM (Gibco) high glucose (4500 mg/L) supplemented with 10% FBS (Sigma Aldrich) and 1% Penicillin Streptomycin (Life Technologies). MCF7 cells were cultured in DMEM low glucose (1500 mg/L) supplemented with 10% FBS and 1% Penicillin Streptomycin. All cells were grown in a cell culture incubator set at 37 °C with 5% CO_2_.

### Constructs

GFP/mCherry Reporter constructs were made first by using pcDNA3 (Invitrogen) as a backbone and inserting PCR amplified eGFP into the EcoRI site downstream of the CMV promoter using In-FusionR Advantage PCR Cloning Kits from Clonetech. The second CMV promoter and mCherry were inserted into the same vector using pcDNA3 eGFP as the starting vector (Molecular Cloning Laboratories, San Francisco).

3′UTR constructs for the control, STIM1, STIM2, Orai1, Orai2 and Orai3 were constructed by MCLab (Molecular Cloning Laboratories, San Francisco). The 3′UTRs were amplified using the following sequences:

1. STIM1 3′UTR was amplified from nucleotides 2945–4356 (NM_001277961.1)
2. STIM2 3′UTR was amplified from nucleotides 2838–5222 (NM_001169117.1).
3. Orai1 3′ UTR was amplified from nucleotides 1100–1496 (NM_032790.3).
4. Orai2 3′UTR was amplified from nucleotides 975–2638 (NM_001126340.3).
5. Orai3 3′UTR was amplified from nucleotides 1111–2204 (NM_152288.3)

Mutations and deletions in the 3′UTR constructs were made using QuikChange II XL Site-Directed Mutagenesis Kit (Agilent Technologies). All mutations were verified by DNA sequencing. Primers used for mutagenesis were as follows:

miR-223 mut:

F: 5′ TCCTTGGGTCCTTCATTATTATTTATTAAATAACCACCATGGCCTGC 3′

R: 5′ GCAGGCCATGGTGGTTATTTAATAAATAATAATGAAGGACCCAAGGA 3′

miR-223 del:

F: 5′ TCATCCTTGGGTCCTTCATTATCACCATGGCCTG 3′

R: 5′ CAGGCCATGGTGATAATGAAGGACCCAAGGATGA 3′

miR-195 mut:

F: 5′ CCCAACCATGGGCTGATACTGTCACTCCCTCTC 3′

R: 5′ GAGAGGGAGTGACAGTATCAGCCCATGGTTGGG 3′

miR-195 del:

F: 5′ CTGCCTCCGTCCCGTCACTCCCTCTC 3′

R: 5′ GAGAGGGAGTGACGGGACGGAGGCAG 3′

miR-150 del:

F: 5′ AGAGGCCAGGCTTGGGAATGGCATCTGTTCAT 3′

R: 5′ ATGAACAGATGCCATTCCCAAGCCTGGCCTCT 3′

miR-326 del:

F: 5′ CCACTCACAGTGGTTCTGTT 3′

R: 5′ TCCTGAGCCCTAGGAGTATTG 3′

miR-183 del:

F: 5′ GGTGGCTAAGGAAGATGGTATAG 3′

R: 5′ AGGGAGTACAAGGCAGTCT 3′

EGFP-hAgo2 (Plasmid #21981)^39^ and pIRESneo-FLAG/HA Ago2 (Plasmid #10822)^40^ were purchased from Addgene.

### Transient transfection

Cells were split 24 hrs before transfection from 80% confluent flasks. HEK-293 and MCF7 cells were transfected using Lipofectamine 2000 (Life Technologies) for plasmid DNA, siRNA and plasmid DNA + siRNA. MDA-MB231 cells were transfected using Lipofectamine LTX (Life Technologies) for plasmid DNA, siRNA and plasmid DNA + siRNA. Ago2 siRNA was obtained from Santa Cruz and Ago2 Trilencer siRNA was obtained from Origene. Pumilio1 and Pumulio2 siRNA were obtained from Santa Cruz Biotechnology.

### Confocal and epifluorescence imaging

Live cell imaging was done by a Zeiss LSM710 confocal using an EC Plan-Neofluar 40×/1.30 oil DIC M27 objective. Imaging was done using the pinhole fully open to capture fluorescence from entire cells. Images were analyzed using ImageJ software. GFP and mCherry reporter assay was used instead of the traditional Luciferase assay for determining the function of the 3′UTR in regulation to be able to observe cells individually instead of a lysed population. GFP over mCherry ratios were obtained for 100 cells per condition and then averaged. This ratio was normalized to the average of the GFP/mCherry ratio of control transfected cells. All experiments were done at least in triplicates.

### Calcium imaging

Transiently transfected HEK 293, MCF7 and MDA-MB231 cells were loaded with 2 μM Fura2-AM (Life Technologies) in DMEM for 30 min at 37 °C. Stores were depleted using 1uM Thapsigargin (Thermo Fisher) in calcium-free Ringer’s solution. 2 mM Calcium was added to the medium after stores were depleted to study SOCE. Imaging was performed with alternating excitation 340-and 380-nm while collecting emission at 510 nm using a PTI imaging system mounted on an Olympus IX71. Calcium imaging was done on different conditions at least in triplicates.

### Western blotting

Cells were lysed in IP lysis buffer as described Srikanth, 2010 #420}. Whole cell lysates were separated on 4–12% Bis-Tris gels (Life Technologies) and proteins were transferred to PVDF membranes (Biorad). Blocking was done using superblock (5% low fat milk, 2% Casein and 1% BSA) or 5% low fat milk Membranes were incubated with the primary antibody in 3% BSA (w/v) in TBST (137 mM NaCl, 20 mM tris, 0.1% Tween-20, pH 7.6) overnight at 4 °C. Membranes were incubated with the HRP-conjugated secondary antibody for one hour at room temperature and bound proteins were visualized using ECL Western blotting detection reagents (GE Healthcare). Blots were imaged using Geliance Gel Imaging system (Perkin Elmer).

Primary antibodies used were STIM1 (4916 S Cell Signaling), Ago2 (2897 S Cell Signaling), β-actin (A1978 from Sigma), Dicer (5362 S Cell Signaling), Drosha (3364 S Cell Signaling), Pumilio1 (12322 S Cell Signaling) and Pumilio2 (12323 S Cell Signaling) Secondary antibodies used were from Jackson ImmunoResearch Laboratories goat anti-rabbit IgG (111-035-044) and goat anti-mouse IgG (115-035-146).

### miRNA quantification and profiling by RT-PCR

Total RNA isolated from the samples were normalized for concentration and equal concentration (5 ng/μl) was used for reverse transcription to cDNA using the miRCURY LNA RT Kit II (Qiagen) according to the manufacturer’s protocol. The miRCURY LNA miRNA QC PCR Panel (Qiagen) was used to assess the quality and integrity of the synthesized cDNA. cDNA was then diluted hundred-fold and mixed 1:1 with 2x miRCURY LNA SYBR Green PCR Kit Master Mix (Qiagen) and ROX Reference Dye (2.5 μl/ml) (ThermoFisher Scientific), before loading onto miRCURY LNA miRNA miRNome PCR Panel, Human panel I + II, V5, 384 well (Qiagen). The panels were subjected to quantitative Real-Time PCR using the QuantStudio 12 K Flex Real-Time PCR System (Applied Biosystems) and the raw data obtained was pre-processed and analyzed using GenEx qPCR analysis software (Version 6, MultiD). The mean value of the UniSp3 inter-plate calibrator was used to carry out inter-plate calibration and account for run-to-run variation among the panels. MicroRNAs with Ct values equal to or exceeding 35 were considered background and left out of the analysis. A no-template negative control was run on the same panels and a ΔCt of 1 between the assay and the negative control was used as a cut-off to eliminate assays with spurious amplifications. A call-rate cut-off of 40% was used to omit microRNA assays that were not present in 60% or more of the samples analyzed. The global mean of all expressed microRNAs with Ct < 35 was used to normalize individual Ct values. MicroRNA assays in which the Ct readings remained undetermined or were represented as “NaN” values, were substituted with the column’s maximum + 1 as per guidelines. Values obtained were first converted to relative quantities on the linear scale and set as relative to nothing, following which log scale conversion was performed. Statistical analyses were done by comparing two groups at a time using an unpaired two-tailed T-test having a Confidence Interval of 95% (P-value ≤ 0.05), along with Bonferroni correction, but advanced multiple testing was not employed.

### miRNA microarray

miRNA microarray was done using the GeneChip miRNA 2.0 Array from Affymetrix. 1 μg total RNA isolated using miRNeasy Mini Kit from Qiagen was used for the FlashTag™ Biotin HSR RNA labeling reaction (Genisphere) followed by poly (A) tailing. Samples were prepared in triplicates for each cell line. These were used for hybridization, washing and scanning with an Affymetrix scanner.

### Northern blot

Total RNA was prepared in triplicates for HEK 293, MDA-MB231 and MCF7 cells using miRNeasy Mini Kit form Qiagen. The RNA concentration was measured using Nanodrop and the quality was checked on a 1% Agarose gel. 20 μg total RNA was used for sample preparation and these were loaded in a 1% Agarose-formaldehyde gel. The gel was run at 80 V for four hours in MOPS running buffer. The gel was washed twice in DEPC-treated H_2_O to remove the formaldehyde. Transfer was done overnight on a Hybond + membrane. The staining was done using standard techniques with a DIG-labeled Stim1 coding probe (1 kb in size), anti-DIG sheep-Fab fragments (Roche) and was developed using NBT/BCIP solution (Roche) overnight in the dark.

### Real Time PCR

Total RNA was purified from Naïve or transfected cells using RNeasy Mini Kit or miRNeasy Mini Kit (Qiagen). First-strand cDNA synthesis was done using SuperScript II (Invitrogen). Quantitative real time PCR was done using transcript specific primers and SYBR Green dye (Applied Biosystems). The samples were analyzed using 7000 Sequence Detection System hardware and software (Applied Biosystems). The specificity of the reactions was determined using melt-curve analysis. β-actin mRNA was used for normalization of the data between samples.

Primers used for RT-PCR were:

1. Stim1 coding primers were obtained from Qiagen (QT00083538)
2. β-actin primers were obtained from Qiagen (QT00095431)
3. Ago2 primer sequences were as follows:

F 5′ TCATGGTCAAAGATGAGATGACAGA 3′

R 5′TTTATTCCTGCCCCCGTAGA 3′

### FACS analysis

GFP or GFP-Ago2 transfected cells were harvested and with Fixable Viability Dyes eFluor 506 (eBioscience) prior to fixation. Cells negatively stained with the dye were live and subjected to further analysis. Fixed and permeabilized cells were then subjected to immunostaining with STIM1 N-terminus Rabbit Polyclonal antibody (Proteintech #11565-1-AP), follow by stained with Goat anti-Rabbit Alexa Fluor 405 secondary antibody (ThermoFisher Scientific). Live GFP^+^ cells were used to analyze endogenous STIM1 expression level. Cells were subjected to a BD Fortessa flow cytometry analyzer (BD Biosciences) and data were analyzed by the FlowJo software (Tree Star).

### Clinical data source

Clinical data were obtained from public datasets available at the Breast Cancer Gene-Expression Miner (v4.1) at the (http://bcgenex.centregauducheau.fr/BC-GEM/GEM-Accueil.php) using their built-in statistical analysis tools described by Jézéquel *et al*.^41,42^.

### Statistics

Data are presented as mean + S.D. with the number of replicas indicated. Statistical analysis for significance were performed using one-way ANOVA, paired t-test, or unpaired t-test as indicated, with significance levels indicated as follows: *p < 0.05; **p < 0.001; ***p < 0.0001; and ns p > 0.05 (not significant).

## Supplementary information

Supplemental Figures
