# Supplementary material for: miRNA-dependent regulation of STIM1 expression in breast cancer
Source: Sci Rep. 2019 Sep 10;9:13076. doi: 10.1038/s41598-019-49629-5 (PMC6736934; doi:10.1038/s41598-019-49629-5)
Supplement: Supplementary file 1 — Supplemental Figures [file 41598_2019_49629_MOESM1_ESM.pdf]

SUPPLEMENTAL MATERIAL

Title: miRNA-dependent regulation of STIM1 expression in breast cancer

Authors:

Rashmi P. Kulkarni
Asha Elmi
Ethel Alcantara-Adap
Satanay Hubrack
Nancy Nader
Fang Yu
Maya Dib
Vimal Ramachandran
Hani Najafi Shoushtari
Khaled Machaca

Supplemental Figures

Supplemental Figure 1

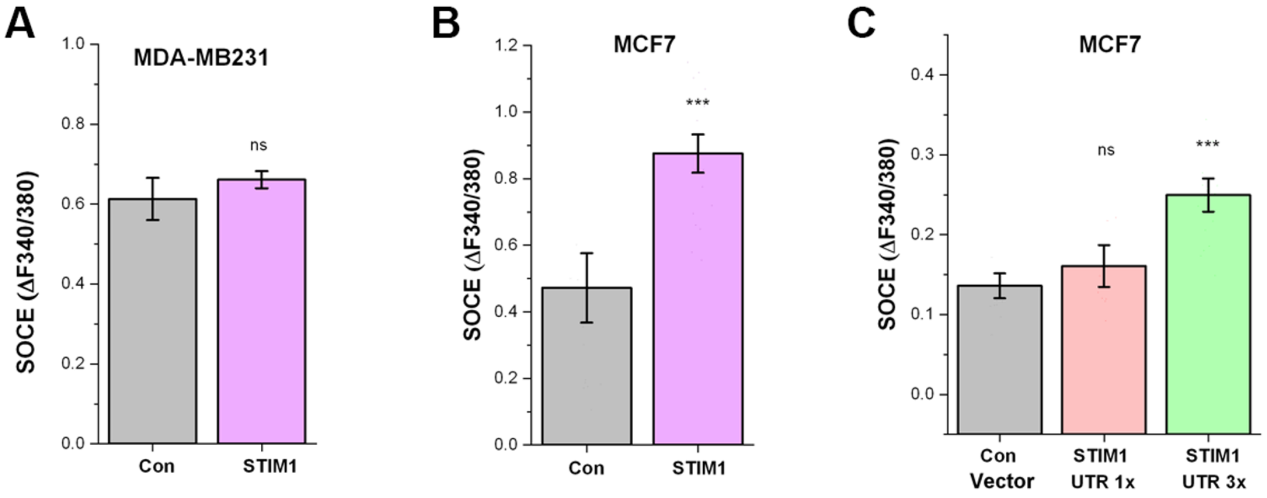

Supplemental Figure 1. The STIM1 3'UTR acts as a miRNA sponge in MCF7 cells.

(**A,B**) SOCE levels in untransfected cells (Con) or cells overexpressing STIM1 in MDA-MB-231 (A; mean ± s.e.m of over 100 cells from 4 different experiments; ANOVA; ns not significant) or MCF7 (B; mean ± s.e.m of over 25 cells from 3 different experiments; ANOVA; *** $p<0.0001$). (**C**) SOCE levels in MCF7 cells transfected with the control GFP-mCherry reporter (Con Vector), or the GFP-mCherry reporter with 1 (1x) or 3 (3x) copies of the STIM1 3'UTR downstream of GFP. Mean ± s.e.m of over 21 cells from 2 different experiments; ANOVA; *** $p<0.0001$; ns not significant).

Supplemental Figure 2

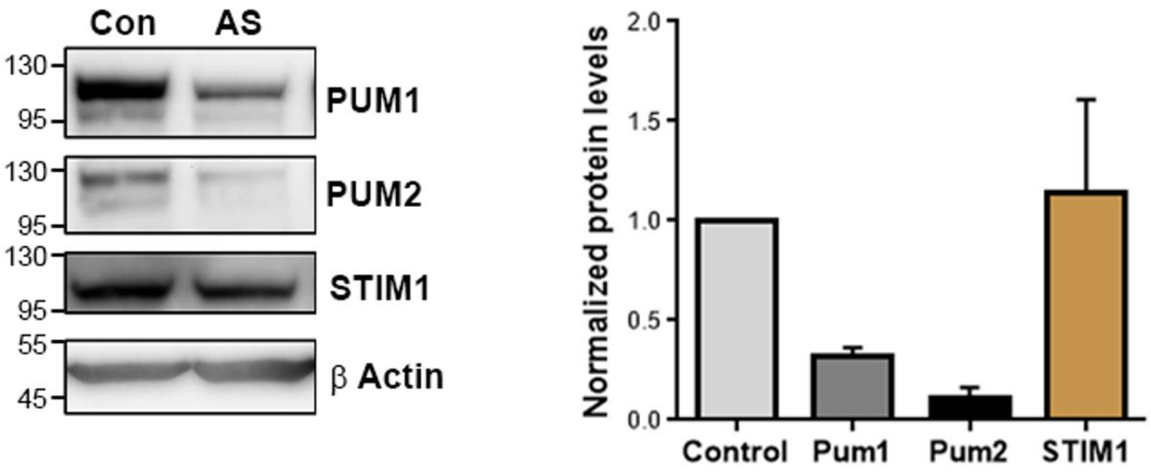

Supplemental Figure 2. Pumilio does not regulate STIM1 expression in MDA-MB-231.

Representative Western blots and summary data of STIM1 expression in control MDA-MB231 cells or in cells transfected with siRNA against Pumilio 1 and 2. The siRNA treatment effectively knocks down Pumilio levels without much effect on STIM1 levels. Actin is used as a loading control and STIM1 protein levels are normalized to actin to minimize variability between different gels (n=3).

Supplemental Figure 3

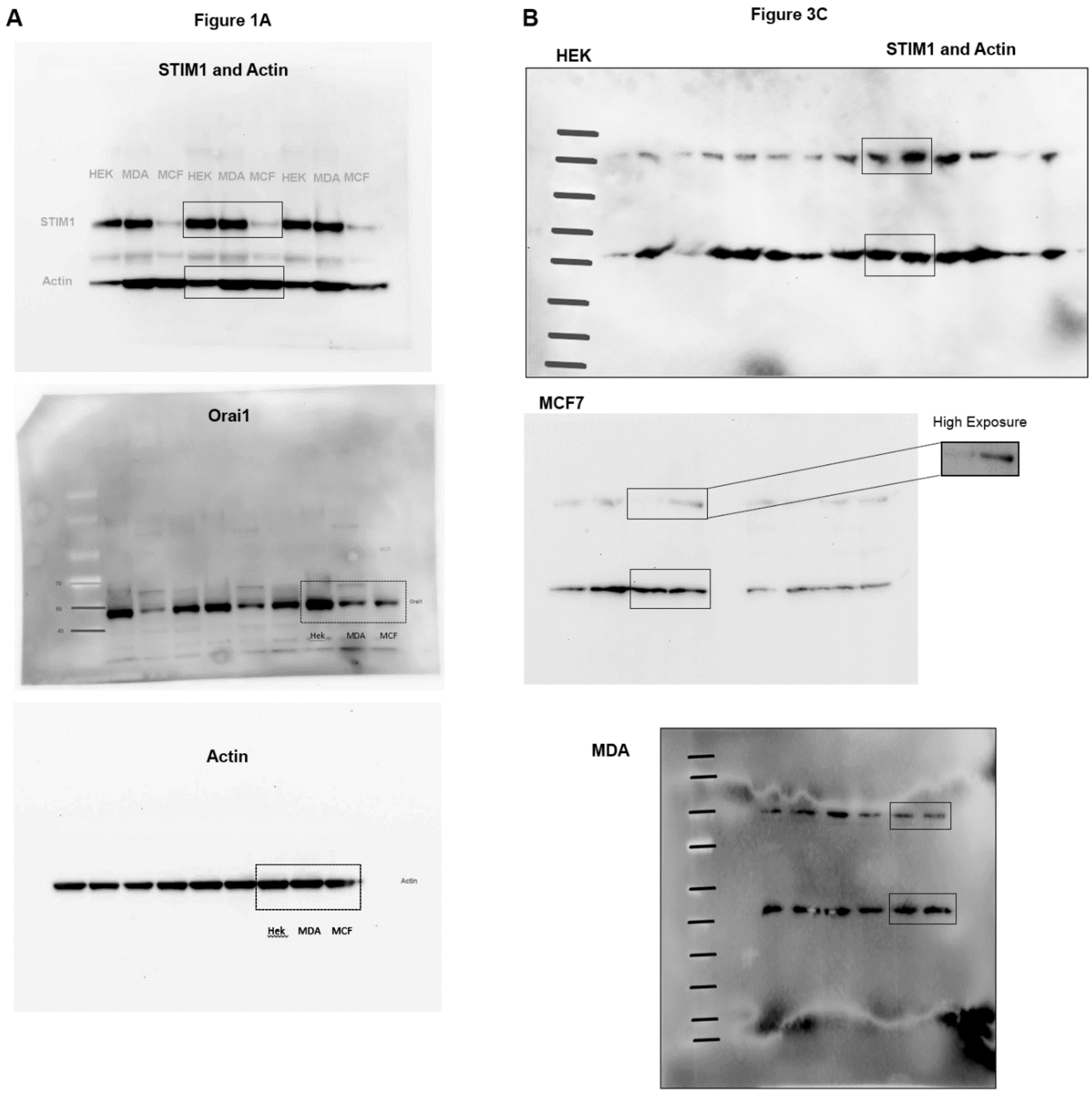

Supplemental Figure 3. Whole blots for Figures 1A and 3C as indicated.

Supplemental Figure 4

A Figure 5B

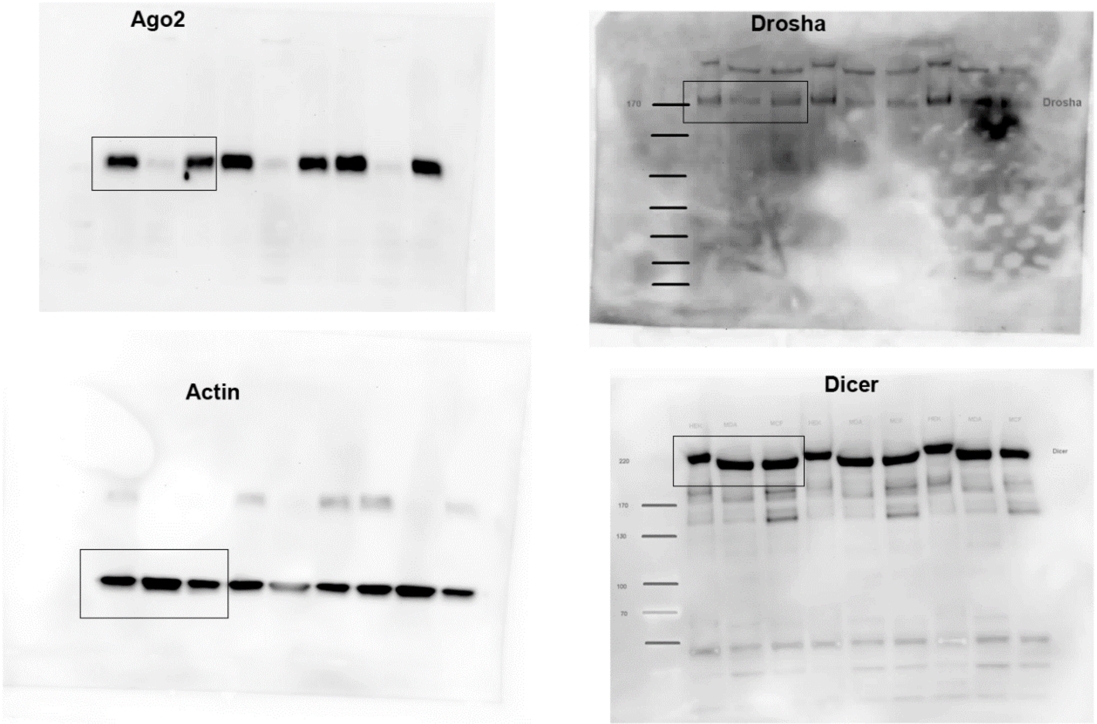

B Figure 5C

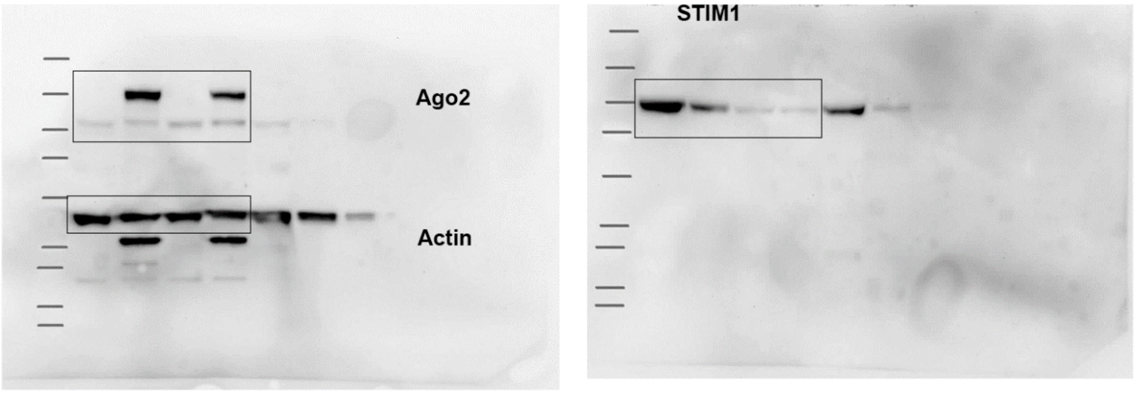

Supplemental Figure 4. Whole blots for Figures 5B and 5C as indicated.

Supplemental Figure 5

A Figure 5D

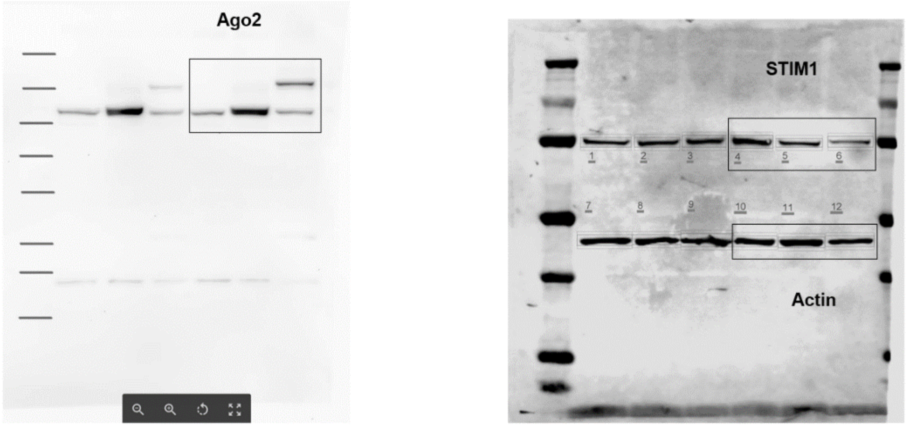

B Supp Figure 2

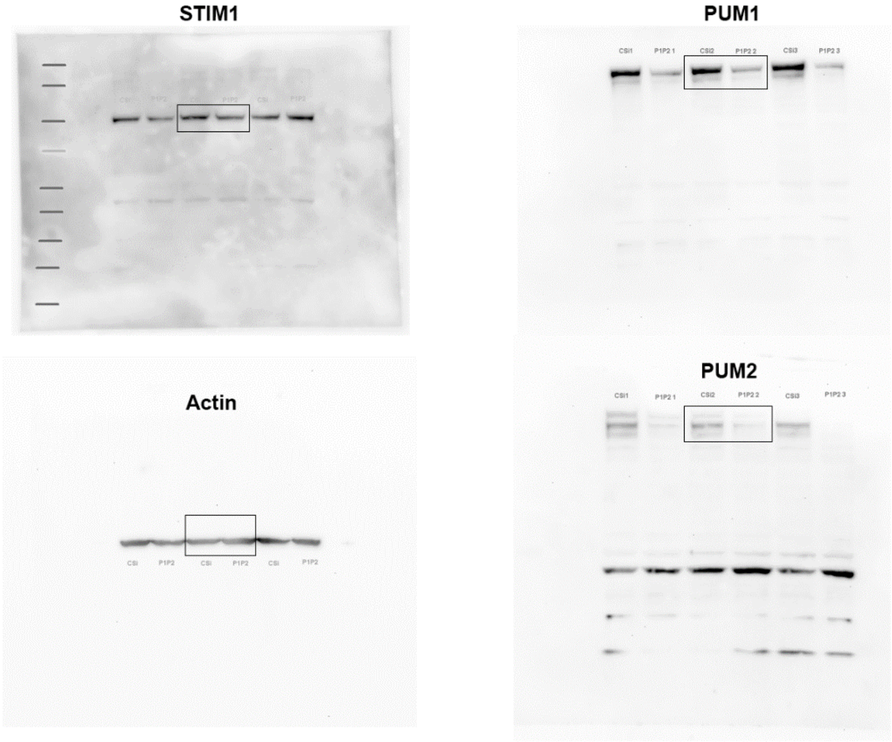

Supplemental Figure 5. Whole blots for Figures 5D and S2 as indicated.
